# Reply to Murphy and Leivada: Program induction can learn language

**DOI:** 10.1073/pnas.2202925119

**Published:** 2022-06-01

**Authors:** Steven T. Piantadosi, Yuan Yang

**Affiliations:** ^a^Department of Psychology, University of California, Berkeley, CA 94720;; ^b^Machine Learning, Georgia Institute of Technology, Atlanta, GA 30332

We present a model that can learn patterns present in natural language (1), a feat long argued to be impossible (2). This is important because the study of learnability helps reveal human nature, potentially pinpointing what is distinctive about human cognition. In this reply, we contest several key points raised by a letter from Murphy and Leivada (3).

They state that our model learns “strings, not structures,” implying that it acquires only sequential properties of its input (“strings”) rather than the structure and hierarchy found in human language (3). Their claim reflects serious misunderstanding: A model that lacks structure cannot, even theoretically, generalize in the way ours does. Our model uses the strings to infer structured, generative processes, building representations that are equivalent to finite-state machines, context-free grammars, and beyond. This is structure learning galore. The model is evaluated by whether it gets the set of strings correct, but this should be uncontroversial. For example, Chomsky (4) begins by defining “a language to be a set (finite or infinite) of sentences.” Gold learnability (2) and associated theories are about getting the string set correct. Just as in doing linguistics, the model gets the string set correct only by inducing the right latent generative structure.

We agree with Murphy and Leivada (3) that children also acquire semantics, and we cite semantic models based on an approach similar to ours. Since Montague, semanticists have formulated theories of compositional meaning that are logical or program-like. Our approach fits within that tradition, as well as within newer work that jointly acquires semantics and syntax using methods akin to program learning. We note that leaving semantics out of our model strengthens our core argument that the generative structures in language are learnable, often easily: Additional semantic information should help learning, so, by excluding it, our work establishes learnability on a harder problem.

With more space, we would contest other points. Murphy and Leivada (3) say the model shows only “moderate success” with a fragment of English, but our estimated F score was 1.0 after a few hundred sentences, which is as high as F scores go. Murphy and Leivada describe our data as unambiguous, but strings are actually hugely ambiguous about the thing we learn—the underlying generative process. This does not prevent the model from working well. Murphy and Leivada point out, as we did, difficulties with English auxiliaries, but they do not mention the provable ability of systems like ours in principle (5). Murphy and Leivada point to noisiness of data without mentioning that we used a noise model which assured some level of robustness. We emphasize that our model is implemented and freely distributed; authors who wish to understand its strengths and limitations can just try it out rather than try to argue on principle.

Children induce remarkable structure throughout their cognitive repertoire (6, 7). It is past time for nativist and empiricist debates in language acquisition to be informed by the remarkable successes of computational learning models that induce latent structure from impoverished data—success which is contrary to decades of theorizing in some linguistic circles.
